# Switching Quantum Interference in Phenoxyquinone Single Molecule Junction with Light

**DOI:** 10.3390/nano10081544

**Published:** 2020-08-06

**Authors:** Abdalghani Daaoub, Sara Sangtarash, Hatef Sadeghi

**Affiliations:** Device Modelling Group, School of Engineering, University of Warwick, Coventry CV4 7AL, UK; Abdalghani.Daaoub@warwick.ac.uk (A.D.); Sara.Sangtarash@warwick.ac.uk (S.S.)

**Keywords:** photochromic molecules, quantum interference, electrical conductance, molecular electronics, photoswitches

## Abstract

Quantum interference (QI) can lead to large variations in single molecule conductance. However, controlling QI using external stimuli is challenging. The molecular structure of phenoxyquinone can be tuned reversibly using light stimulus. In this paper, we show that this can be utilized to control QI in phenoxyquinone derivatives. Our calculations indicate that, as a result of such variation in molecular structure of phenoxyquinone, a crossover from destructive to constructive QI is induced. This leads to a significant variation in the single molecule conductance by a couple of orders of magnitude. This control of QI using light is a new paradigm in photosensitive single molecule switches and opens new avenues for future QI-based photoswitches.

## 1. Introduction

Studying charge transport through molecular devices has attracted a growing interest in the last decade [1,2,3]. In particular, understanding the influence of light on properties of single molecular-scale devices can have applications on better characterization of the molecular junctions e.g., Raman spectroscopy or used as an alternative to a molecular scale field effect transistor [4,5,6]. In single molecule optoelectronics, one of the main motivations is to switch electrical conductance on and off using an external stimulus such as an external electric field [7,8], redox chemistry [9], or light [4,5,10,11]. In the latter, the electrical current is switched reversibly on and off in photochromic molecules in the presence of light or a change in its intensity. These can be mediated by electron transfers, photo-induced bond cleavages, pericyclic reactions, *E–Z* isomerizations or intra-molecular hydrogen/group transfer [4,12,13,14,15,16,17]. In *E–Z* isomerizations, the structure of molecule changes upon irradiation with light at specific wavelengths [18]. For example, the molecular structure of azobenzene [19] and stilbene [20] changes from *trans* to *cis* confirmation by ultraviolet light [10]. This leads to changes in the electrical conductance of the molecule between two electrodes. The conductance measurement of azobenzene between metallic electrodes shows that the single molecule conductance of *cis* confirmation is higher than *trans* confirmation by only a factor of two in agreement with predictions from theory (see Section A of the supporting information SI and Figures S1 and S2) [21,22]. This ratio is higher in self-assembled monolayers formed by azobenzene molecules because of changes in contacting modality and molecular film thickness [23,24]. However, these ratios are not sufficient to deliver high efficiency photoswitches.

Quantum interference (QI) can lead to a large variation (e.g., couple of orders of magnitude) in single molecule conductance as demonstrated in several molecular junctions [25]. For example, by simply changing the connection point to electrodes from *para* to *meta* in oligo(phenylene-ethynylenes) OPE3, the single molecule conductance changes by more than 30 times at room temperature due to a crossover from a destructive to constructive QI [26]. However, in-situ chemical modification of a molecule in the junction using external stimulus is challenging.

**Figure 1:**
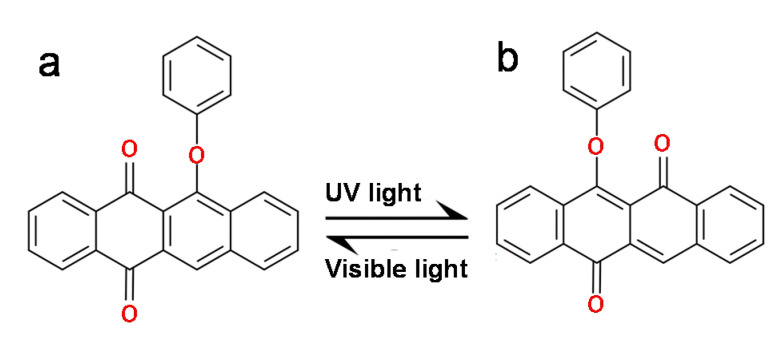
Chemical structures of (**a**) *trans* and (**b**) *cis* phenoxyquinone isomers. In visible light, phenoxyquinone molecule takes *trans* configuration, whereas, under UV light, it is changed to *cis* confirmation.

In what follows, our aim is to demonstrate that QI in a single molecule junction can be modulated from destructive to constructive in phenoxyquinone photosensitive molecules. *Trans* and *cis* configurations of phenoxyquinone are shown in Figure 1. These isomers are stable under different light stimuli [27]. In visible light, phenoxyquinone molecule takes *trans* configuration (Figure 1a) whereas under UV light it is changed to *cis* (Figure 1b) configuration [28]. The energy needed for this transition is c.a. 300 *meV* [27]. This process is reversible and *cis* isomer can be transformed to *trans* isomer through irradiation or heating with visible light [29]. This makes phenoxyquinone derivatives attractive candidates for photoswitching. Here, we demonstrate for the first time that this light induced change in molecular structure controls room temperature QI in of phenoxyquinone molecules and a crossover from destructive QI (DQI) to constructive QI (CQI) takes place. This is significant because such crossover from CQI to DQI does not take place in other photochromic molecules such as azobenzene or dithienylethene leading to smaller on/off ratio. The crossover from CQI to DQI in phenoxyquinone leads to a couple of orders of magnitude increase in electrical current and a large on/off ratio of several orders of magnitude is accessible.

## 2. Computational Methods

The Hamiltonian of the structures described in this paper was obtained using DFT as described below or constructed from a simple tight-binding model with a single orbital per atom. The optimized geometry and ground-state Hamiltonian and overlap matrix elements of each structure were self-consistently obtained using the SIESTA [30] implementation of density functional theory DFT. SIESTA employs norm-conserving pseudopotentials to account for the core electrons and linear combinations of atomic orbitals to construct the valence states. The generalized gradient approximation (GGA) of the exchange and correlation functional is used with the Perdew–Burke–Ernzerhof parameterization, a double-ζ polarized basis set, and a real space grid defined with an equivalent energy cutoff of 250 *Ry*. The geometry optimization for each structure is performed for the forces smaller than 40 *meV/Å*.

The mean-field Hamiltonian obtained from the converged DFT calculation or a simple tight-binding Hamiltonian was combined with Gollum [31,32] implementation of the non-equilibrium Green’s function method to calculate the phase-coherent, elastic scattering properties of the each system consist of left (source) and right (drain) leads and the scattering region. The transmission coefficient T(E)  for electrons of energy E (passing from the source to the drain) is calculated via the relation $T\left(E\right)=trace\left(\Gamma_{R}\left(E\right)G^{R}\left(E\right)\Gamma_{L}\left(E\right)E^{R^{†}}\left(E\right)\right)$. In this expression,$\;\Gamma_{L,R}\left(E\right)=i\left(\;\underset{L,R}{\sum }\left(E\right)-\overset{†}{\underset{L,R}{\sum }}\left(E\right)\right)$ describes the level broadening due to the coupling between left (L) and right (R) electrodes and the central scattering region, $\Sigma_{L,R}\left(E\right)$ are the retarded self-energies associated with this coupling, and $G_{R}={\left(ES-H-\Sigma_{L}-\Sigma_{R}\right)}^{-1}$ is the retarded Green’s function, where *H* is the Hamiltonian and *S* is overlap matrix. Using the obtained transmission coefficient  T(E), the electrical current I and conductance G could be calculated by the Landauer formula $I=\frac{e}{h}\int^{\;}dE\;T\left(E\right)\left(f\left(eV/2\right)-f\left(-eV/2\right)\right)$$G=G_{0\;}\int^{\;}dE\;T\left(E\right)\left(-\frac{\partial f}{\partial E}\right),$ where $G_{0}=2e^{2}/h$ is the conductance quantum, *V* is bias voltage, and *f* is Fermi distribution function. At low temperatures (e.g., T = 0K), Landauer formula simplifies to $G=G_{0\;}T\left(E_{F}\right)$.

## 3. Results

**Figure 2:**
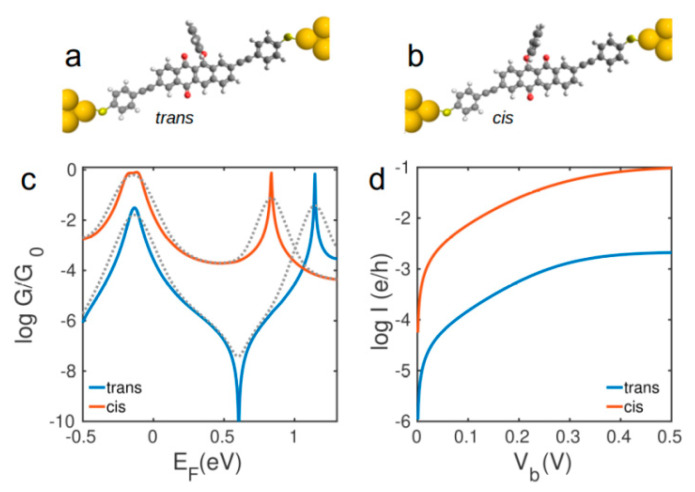
Molecular structures of (**a**) *trans* and (**b**) *cis* isomers of phenoxyquinone with thiol anchor connected to gold electrodes, (**c**) low temperature conductance and (**d**) room temperature current for (**a**,**b**). Dashed lines in (**c**) show the corresponding room temperature conductances.

**Figure 3:**
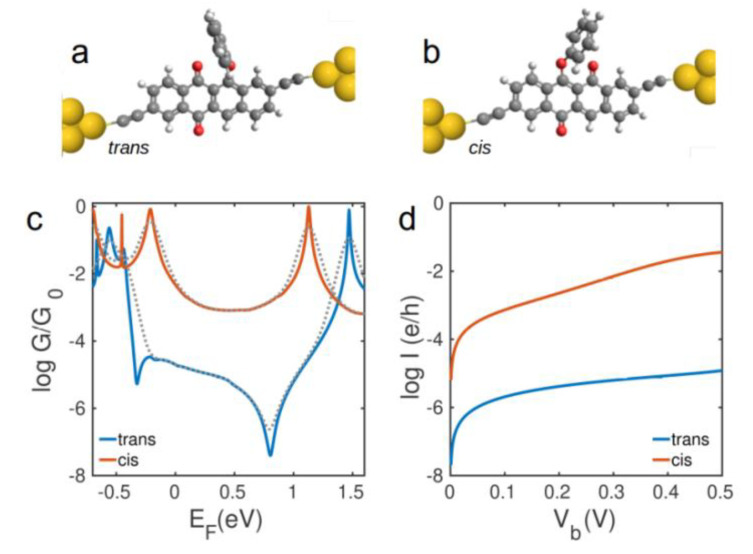
Molecular structures of (**a**) *trans* and (**b**) *cis* isomers of phenoxyquinone with acetylene linker and direct Au-C connection to gold electrodes; (**c**) low temperature conductance and (**d**) room temperature current for (**a**,**b**). Dashed lines in (**c**) show the corresponding room temperature conductances.

We use density functional theory (DFT) to obtain ground state geometry of *cis* and *trans* phenoxyquinone isomers (see method section). Our calculation shows that the ground state energy of *trans* isomer is 310 *meV* lower than *cis* isomer in agreement with reported experimental values [27]. The highest occupied molecular orbital (HOMO) and lowest unoccupied molecular orbital (LUMO) are shown in Figure S3 of the SI. Clearly, the HOMO-LUMO energy gap of *trans*-phenoxyquinone (2.02 *eV*) is 0.5 *eV* larger than *cis*-phenoxyquinone (1.52 *eV*). To study electron transport properties of phenoxyquinone derivatives, we use two different anchor groups, thiol and direct Au-C bond to attach molecules to gold electrodes (Figure 2 and Figure 3). We then obtain the ground state mean field Hamiltonian from optimized geometry of each structure using DFT. The transmission probability [31] *T*(*E*) of electrons with energy *E* traversing through each junction is then calculated. The electrical conductance *G* = *G_0_T*(*E_F_*) and current *I* are then obtained from Landauer formula (see methods). Note that room temperature electrical conductance is obtained from thermal averaging of transmission function using Fermi distribution.

Figure 2a,b show the molecular junctions formed by *trans* and *cis* phenoxyquinone molecules using thiol anchor, respectively. The electrical conductance of *trans*-phenoxyquinone is shown by a blue curve in Figure 2c. The dip in *G*(*E_F_*) is due to destructive quantum interference which leads to a low electrical conductance in *trans* isomer (dashed lines show the room temperature electrical conductance). In contrast, constructive interference through *cis*-phenoxyquinone leads to a higher conductance. At DFT Fermi energy (*E_F_* = 0 *eV*), the conductance is 8 × 10^−3^ *G_0_* for *cis* and 3 × 10^−5^ *G_0_* for *trans* isomers. Clearly, the conductance of *cis*-phenoxyquinone is at least two orders of magnitude higher than that of *trans*-phenoxyquinone not only at *E_F_* = 0 *eV,* but also for whole energy range between HOMO and LUMO of *trans*-phenoxyquinone (Figure 2c). Figure 2d shows the room temperature electrical current through phenoxyquinone for different bias voltages between two electrodes (Figure 2a,b). The current is more than two orders of magnitude larger in *cis* isomer compared to that of *trans* isomer. The transition from low conductance *trans* isomer (off state) which can be induced by UV light to high conductance *cis* isomer (on state) leads to an on/off ratio of > 100. This large variation in electrical conductance from *cis* to *trans* phenoxyquinone demonstrates the potential of these molecular junctions for photoswitching application.

To demonstrate that this large variation in electrical conductance is independent of the choice of the anchor group to electrodes, we calculate electron transport through phenoxyquinone derivatives using direct coupling to the gold electrodes (Au-C bond) as shown in Figure 3a,b. Figure 3c shows the conductance *G*(*E_F_*) of junctions of Figure 3a,b. Clearly, the electron transmission through *trans*-phenoxyquinone is governed by DQI leading to a low electrical conductance, whereas electrons transmit through *cis*-phenoxyquinone junction constructively (high conductance). The conductance values are 1 × 10^−2^ *G_0_* and 2 × 10^−4^ *G_0_* for *cis* and *trans* isomers, respectively, at *E_F_* = 0 *eV*. The electrical conductance through *cis*-phenoxyquinone is 2–5 orders of magnitude higher than *trans*-phenoxyquinone for a wide energy range in the vicinity of *E_F_* = 0 *eV*, meaning that, regardless of the Fermi energy of electrodes, the electrical conductance of *cis* isomer is much higher than that of *trans* isomer. Figure 3d shows the electrical current for junctions of Figure 3a,b for different bias voltages at room temperature. Clearly, a large on/off ratio of > 1000 is accessible at *V_b_* > 0.45 *V*. This is higher than the on/off ratio of dithienylethene, azobenzene, and stilbene photochromic molecules studied between gold or graphene electrodes [10,11,15,33] and is a consequence of crossover from DQI to CQI in phenoxyquinone molecules.

**Figure 4:**
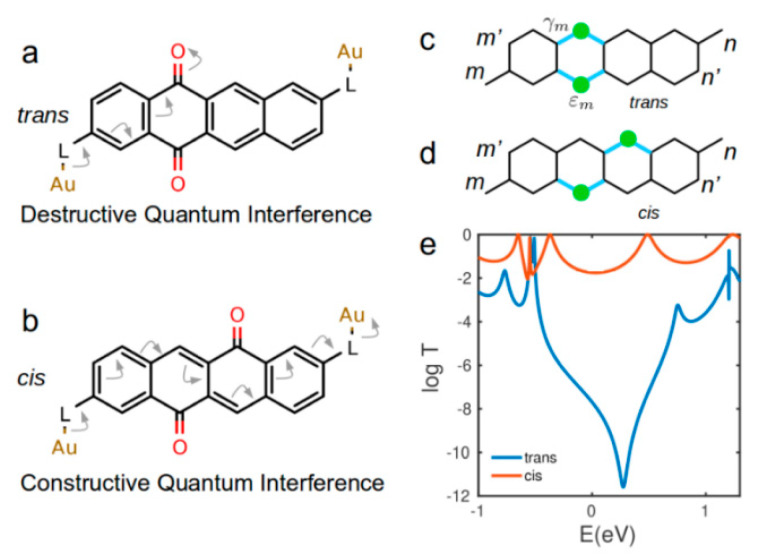
QI based on curly arrows rules and tight binding model. Arrow-pushing pathways for (**a**) *cis* and (**b**) *trans* phenoxyquinone isomers. (**c**,**d**) simplified tight binding picture and (**e**) electron transmission coefficient curves for *trans* and *cis* isomers as a function of electron energy for tight-binding model of site energy *ε_0_ =* 0 and nearest-neighbor couplings *γ* = −1. On-site energy at red sites (Figure 4) is *ε_m_* = −0.55*γ* and *γ_m_* = −0.15.

This large variation in the electrical conductance is due to a crossover from conjugated *cis*- phenoxyquinone to cross conjugated *trans*-phenoxyquinone. This can be understood using curly arrows rules [34]. Arrow-pushing pathways are ‘dead ends’ in *trans* configuration (Figure 4a), whereas there is a ‘pathway’ from one electrode to the other in *cis* configuration (Figure 4b). This means that, from curly arrows rules, a DQI (CQI) is expected for *cis* (*trans*) isomer in agreement with DFT results in Figure 2 and Figure 3. The oxygen atom in carbonyl group (C=O) acts like scissors which weaken the electronic coupling between the neighbouring carbon atom (shown by green dots in Figure 4c,d) with its other neighbouring atoms (shown with blue coupling in Figure 4c,d). The oxygen atom in carbonyl group also changes on-site energy of neighbouring carbon atoms by modifying the charge on the carbon atom. Using this picture, we build a simple single orbital per atom tight binding model as shown in Figure 4 (see methods for details). We then calculate transmission coefficient *T*(*E*) between site *m* and *n* (Figure 4c,d) for both *trans* and *cis* isomers as shown in Figure 4e. We obtain DQI in *trans* isomer in contrast to CQI in *cis* isomer (Figure 4e) in qualitative agreement with our material specific DFT result (Figure 2 and Figure 3).

In order to demonstrate the effect of connection point to the electrodes on QI through *trans* and *cis* configurations of phenoxyquinone, we also calculate transmission coefficient between (*m*, *n’*), (*m’*, *n*) and (*m’*, *n’*) connectivities (Figure 4c,d) using material specific Hamiltonians obtained from DFT. We obtain constructive interference for both *cis* and *trans* isomers when they connected to the electrodes from (*m*, *n’*) and (*m’*, *n*) connectivities (Figure S5a–f of the Supplementary Materials), whereas quantum interference is destructive for both *cis* and *trans* isomers through (*m’*, *n’*) connectivity (Figure S5g–i of the Supplementary Materials). This shows that the manifestation of light induced QI depends on the connection point of phenoxyquinone core to the electrodes. This is also in agreement with our simple tight-binding model (Figures S5j–l and S7j–l of the Supplementary Materials). Using different anchor groups or electrode configuration does not change the result as shown in Figures S6 and S7 of the Supplementary Materials.

## 4. Conclusions

In summary, photochromic molecules change their conformation when exposed to ultraviolet or visible light irradiation. In this paper, we demonstrated that such conformational change in phenoxyquinone derivatives can lead to a significant variation in their single molecule conductance by a couple of orders of magnitude. We attributed this to a reversible crossover from destructive interference in *trans* isomer to constructive interference in *cis* isomer, which leads to a large variation in current (>1000 times) at room temperature.

## Supplementary Materials

The following are available online at https://www.mdpi.com/2079-4991/10/8/1544/s1, Figure S1. Frontier molecular orbitals of azobenzene. Figure S2. Transport through of azobenzene derivatives. Figure S3. Frontier molecular orbitals of phenoxyquinone. Figure S4. Energy and isosurfaces of phenoxyquinone molecular orbitals for *trans* and *cis* isomers. Figure S5. Molecular structure of the junctions formed phenoxyquinone isomers and electron transport through the junctions for different connectivities to electrodes. Figure S6. Molecular structure of the junctions formed phenoxyquinone isomers and electron transport through the junctions using different anchor / electrode configuration. Figure S7. Molecular structure of the junctions formed phenoxyquinone isomers and electron transport through the junctions for different connectivities to electrodes using direct Au-C.

## References

[1] Seong H.C., Kim B., Frisbie C.D. (2008). Electrical resistance of long conjugated molecular wires. Science.

[2] Reed M.A., Zhou C., Muller C.J., Burgin T.P., Tour J.M. (1997). Conductance of a molecular junction. Science.

[3] Tao N.J. (2006). Electron transport in molecular junctions. Nat. Nanotechnol..

[4] Russew M.-M., Hecht S. (2010). Photoswitches: From Molecules to Materials. Adv. Mater..

[5] Goulet-Hanssens A., Eisenreich F., Hecht S. (2020). Enlightening Materials with Photoswitches. Adv. Mater..

[6] Chikkaraddy R., De Nijs B., Benz F., Barrow S.J., Scherman O.A., Rosta E., Demetriadou A., Fox P., Hess O., Baumberg J.J. (2016). Single-molecule strong coupling at room temperature in plasmonic nanocavities. Nature.

[7] Harzmann G.D., Frisenda R., Van Der Zant H.S.J., Mayor M. (2015). Single-Molecule Spin Switch Based on Voltage-Triggered Distortion of the Coordination Sphere. Angew. Chem. Int. Ed..

[8] Zhang J.L., Zhong J.Q., Lin J.D., Hu W.P., Wu K., Xu G.Q., Wee A.T.S., Chen W. (2015). Towards single molecule switches. Chem. Soc. Rev..

[9] Canary J.W. (2009). Redox-triggered chiroptical molecular switches. Chem. Soc. Rev..

[10] Zhang X., Hou L., Samorì P. (2016). Coupling carbon nanomaterials with photochromic molecules for the generation of optically responsive materials. Nat. Commun..

[11] Huang X., Li T. (2020). Recent progress in the development of molecular-scale electronics based on photoswitchable molecules. J. Mater. Chem. C.

[12] Bouas-Laurent H., Dürr H. (2001). Organic photochromism (IUPAC Technical Report). Pure Appl. Chem..

[13] Jia C., Wang J., Yao C., Cao Y., Zhong Y., Liu Z., Liu Z., Guo X. (2013). Conductance switching and mechanisms in single-molecule junctions. Angew. Chem. Int. Ed..

[14] Kim Y., Hellmuth T.J., Sysoiev D., Pauly F., Pietsch T., Wolf J., Erbe A., Huhn T., Groth U., Steiner U.E. (2012). Charge transport characteristics of diarylethene photoswitching single-molecule junctions. Nano Lett..

[15] Dulić D., van der Molen S.J., Kudernac T., Jonkman H.T., de Jong J.J.D., Bowden T.N., van Esch J., Feringa B.L., van Wees B.J. (2003). One-way optoelectronic switching of photochromic molecules on gold. Phys. Rev. Lett..

[16] Marquardt C.W., Grunder S., Błaszczyk A., Dehm S., Hennrich F., Löhneysen H.V., Mayor M., Krupke R. (2010). Electroluminescence from a single nanotube-molecule-nanotube junction. Nat. Nanotechnol..

[17] Roldan D., Kaliginedi V., Cobo S., Kolivoska V., Bucher C., Hong W., Royal G., Wandlowski T. (2013). Charge transport in photoswitchable dimethyldihydropyrene-type single-molecule junctions. J. Am. Chem. Soc..

[18] Purkait M.K., Sinha M.K., Mondal P., Singh R. (2018). Photoresponsive Membranes. Interface Sci. Technol..

[19] Rau H. (2002). Photoisomerization of Azobenzenes. Photoreactive Organic Thin Films.

[20] Martin S., Haiss W., Higgins S.J., Nichols R.J. (2010). The Impact of E—Z Photo-Isomerization on Single Molecular Conductance. Nano Lett..

[21] Qin M., Huang Y., Li F., Song Y. (2015). Photochromic sensors: A versatile approach for recognition and discrimination. J. Mater. Chem. C.

[22] Osella S., Samorì P., Cornil J. (2014). Photoswitching Azobenzene Derivatives in Single Molecule Junctions: A Theoretical Insight into the I/V Characteristics. J. Phys. Chem. C.

[23] Mativetsky J.M., Pace G., Elbing M., Rampi M.A., Mayor M., Samorì P. (2008). Azobenzenes as Light-Controlled Molecular Electronic Switches in Nanoscale Metal—Molecule—Metal Junctions. J. Am. Chem. Soc..

[24] Kumar A.S., Ye T., Takami T., Yu B.C., Flatt A.K., Tour J.M., Weiss P.S. (2008). Reversible photo-switching of single azobenzene molecules in controlled nanoscale environments. Nano Lett..

[25] Xiang D., Wang X., Jia C., Lee T., Guo X. (2016). Molecular-Scale Electronics: From Concept to Function. Chem. Rev..

[26] Liu X., Sangtarash S., Reber D., Zhang D., Sadeghi H., Shi J., Xiao Z.Y., Hong W., Lambert C.J., Liu S.X. (2017). Gating of Quantum Interference in Molecular Junctions by Heteroatom Substitution. Angew. Chem. Int. Ed..

[27] Park I.S., Heo E.J., Kim J.M. (2011). A photochromic phenoxyquinone based cyanide ion sensor. Tetrahedron Lett..

[28] Pace G., Ferri V., Grave C., Elbing M., von Hanisch C., Zharnikov M., Mayor M., Rampi M.A., Samori P. (2007). Cooperative light-induced molecular movements of highly ordered azobenzene self-assembled monolayers. Proc. Natl. Acad. Sci. USA.

[29] Moresco F., Meyer G., Rieder K.-H., Tang H., Gourdon A., Joachim C. (2001). Conformational Changes of Single Molecules Induced by Scanning Tunneling Microscopy Manipulation: A Route to Molecular Switching. Phys. Rev. Lett..

[30] Soler J.M., Artacho E., Gale J.D., García A., Junquera J., Ordejón P., Sánchez-Portal D. (2002). The SIESTA method for ab initio order- N materials simulation. J. Phys. Condens. Matter..

[31] Sadeghi H. (2018). Theory of electron, phonon and spin transport in nanoscale quantum devices. Nanotechnology.

[32] Ferrer J., Lambert C.J., García-Suárez V.M., Manrique D.Z., Visontai D., Oroszlany L., Rodríguez-Ferradás R., Grace I., Bailey S.W.D., Gillemot K. (2014). GOLLUM: A next-generation simulation tool for electron, thermal and spin transport. New J. Phys..

[33] Jia C., Migliore A., Xin N., Huang S., Wang J., Yang Q., Wang S., Chen H., Wang D., Feng B. (2016). Covalently bonded single-molecule junctions with stable and reversible photoswitched conductivity. Science.

[34] Kermack W.O., Robinson R. (1922). LI.—An explanation of the property of induced polarity of atoms and an interpretation of the theory of partial valencies on an electronic basis. J. Chem. Soc. Trans..

