# Supplementary material for: Switching Quantum Interference in Phenoxyquinone Single Molecule Junction with Light
Source: Nanomaterials (Basel). 2020 Aug 6;10(8):1544. doi: 10.3390/nano10081544 (PMC7466391; doi:10.3390/nano10081544)
Supplement: Supplementary file 1 [file nanomaterials-10-01544-s001.pdf]

nanomaterials

Supporting information

Switching Quantum Interference in Phenoxyquinone Single Molecule Junction with Light

Abdalghani Daaoub, Sara Sangtarash and Hatef Sadeghi *

Device Modelling Group, School of Engineering, University of Warwick, CV4 7AL Coventry, UK; Abdalghani.Daaoub@warwick.ac.uk (A.D.); Sara.Sangtarash@warwick.ac.uk (S.S.)
*Correspondence: Hatef.Sadeghi@warwick.ac.uk

A-Azobenzene photoswitches

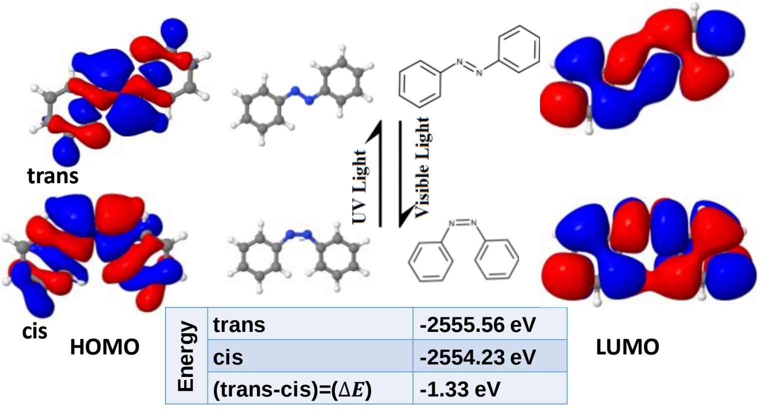

Energy		
	trans	-2555.56 eV
	cis	-2554.23 eV
	(trans-cis)=(ΔE)	-1.33 eV

Figure S1. Frontier molecular orbitals of Azobenzene. HOMO and LOMO orbital for both *trans* and *cis* Azobenzene isomers. The ground state energy of *trans* isomer is higher than *cis* by 0.55 *eV*.

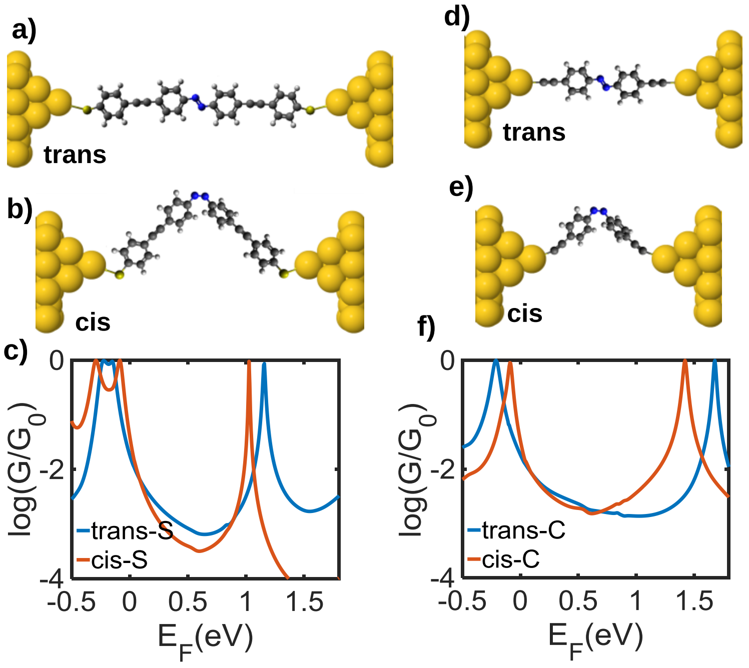

Figure S2. Transport through of Azobenzene derivatives. Molecular structures of (**a**,**d**) *trans* and (**b**,**e**) *cis* isomers of Azobenzene molecules connected to the gold electrodes through thiol and acetylene anchor groups. DFT conductance versus electrode Fermi energies for *trans* and *cis* isomers (**c**) with thiol anchor and (**f**) with acetylene linker.

B-Phenoxyquinone photoswitches

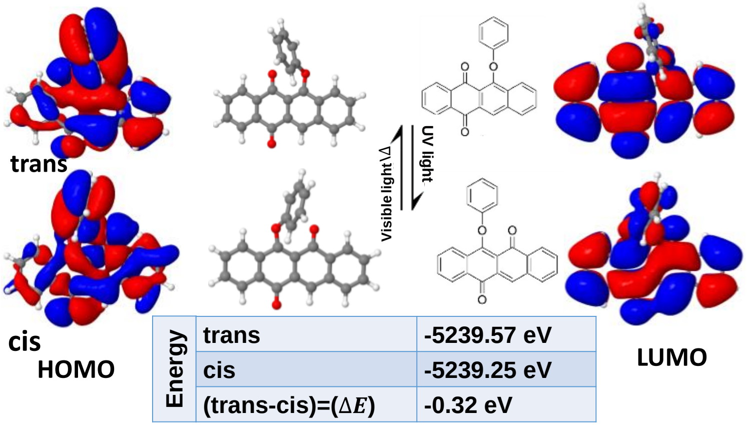

Figure S3. Frontier molecular orbitals of phenoxyquinone. HOMO and LOMO orbital for both *trans* and *cis* phenoxyquinone isomers. The ground state energy of *trans* isomer is higher than *cis* by 0.32 eV.

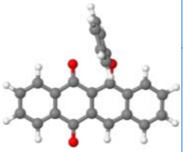

Trans	HOMO-2	HOMO-1	HOMO	LUMO	LUMO+1	LUMO+2
Energy (eV)	-5.22	-4.95	-4.87	-2.85	-2.38	-1.23
Wave function	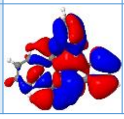	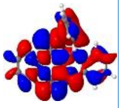	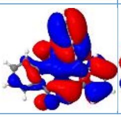	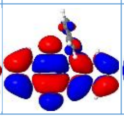	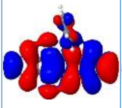	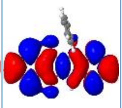

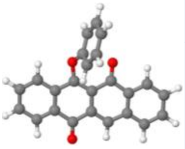

Cis	HOMO-2	HOMO-1	HOMO	LUMO	LUMO+1	LUMO+2
Energy(eV)	-5.11	-4.75	-4.69	-3.17	-2.08	-1.18
Wave function	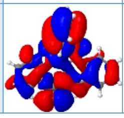	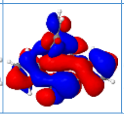	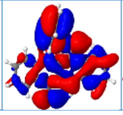	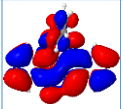	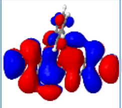	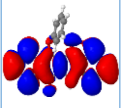

Figure S4. Energy and isosurfaces of phenoxyquinone molecular orbitals for trans and cis isomer.

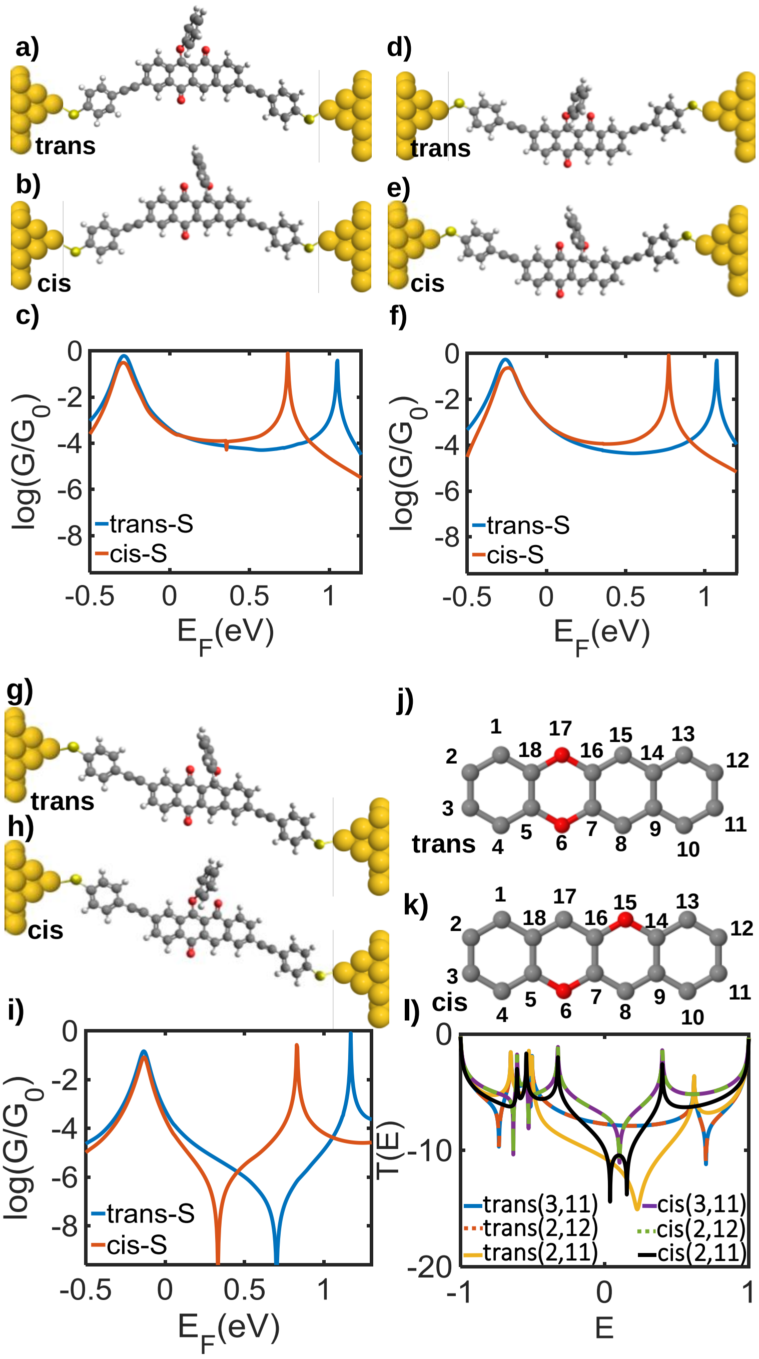

Figure S5. Molecular structure of the junctions formed phenoxyquinone isomers and electron transport through the junctions for different connectivities to electrodes. (**a–f**) through *meta* connectivities (**g–j**) through *para* connectivity. (**j–l**) results obtained using simple tight binding model.

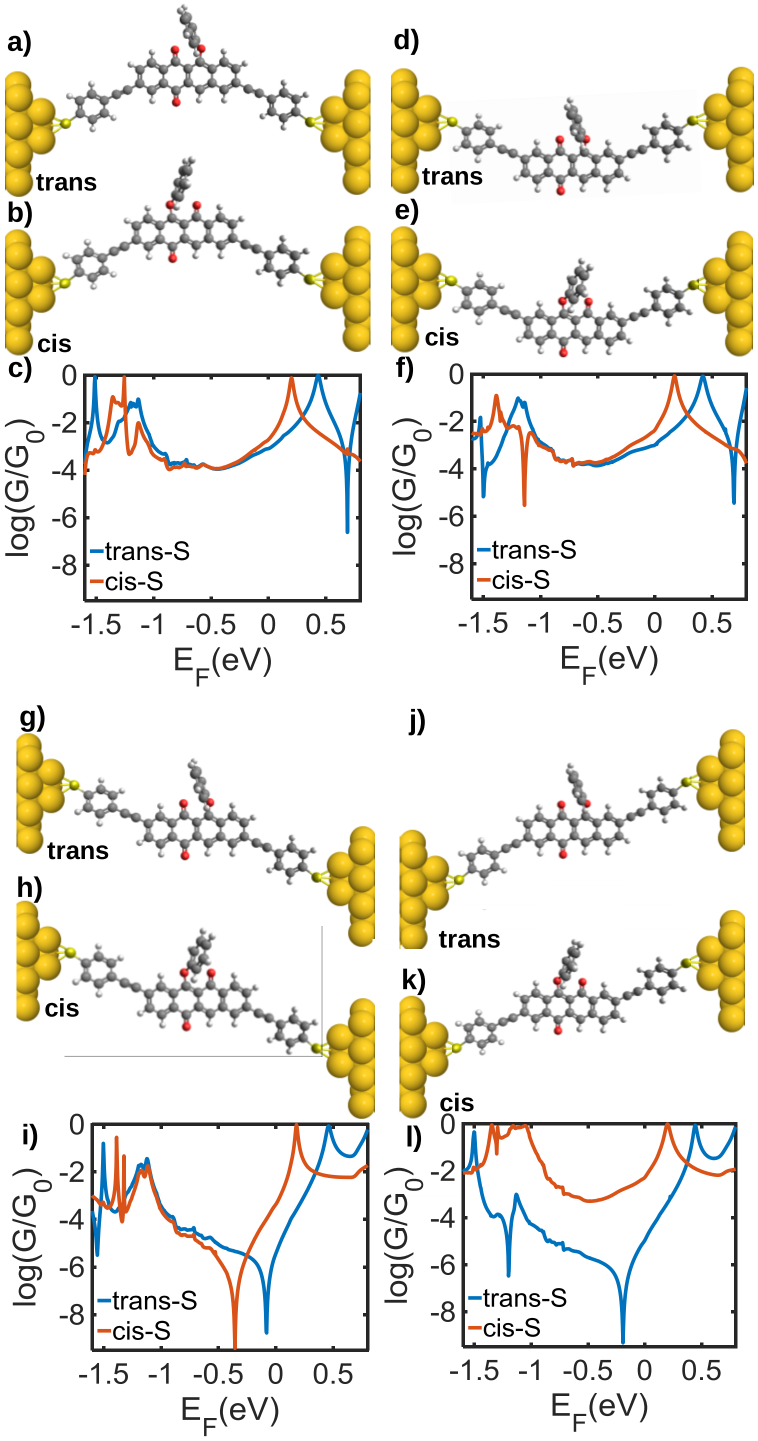

Figure S6. Molecular structure of the junctions formed phenoxyquinone isomers and electron transport through the junctions using different anchor / electrode configuration. These results are in qualitative agreement with those of obtained using tip electrodes shown in Figure 2 of the main text and S4 of the SI.

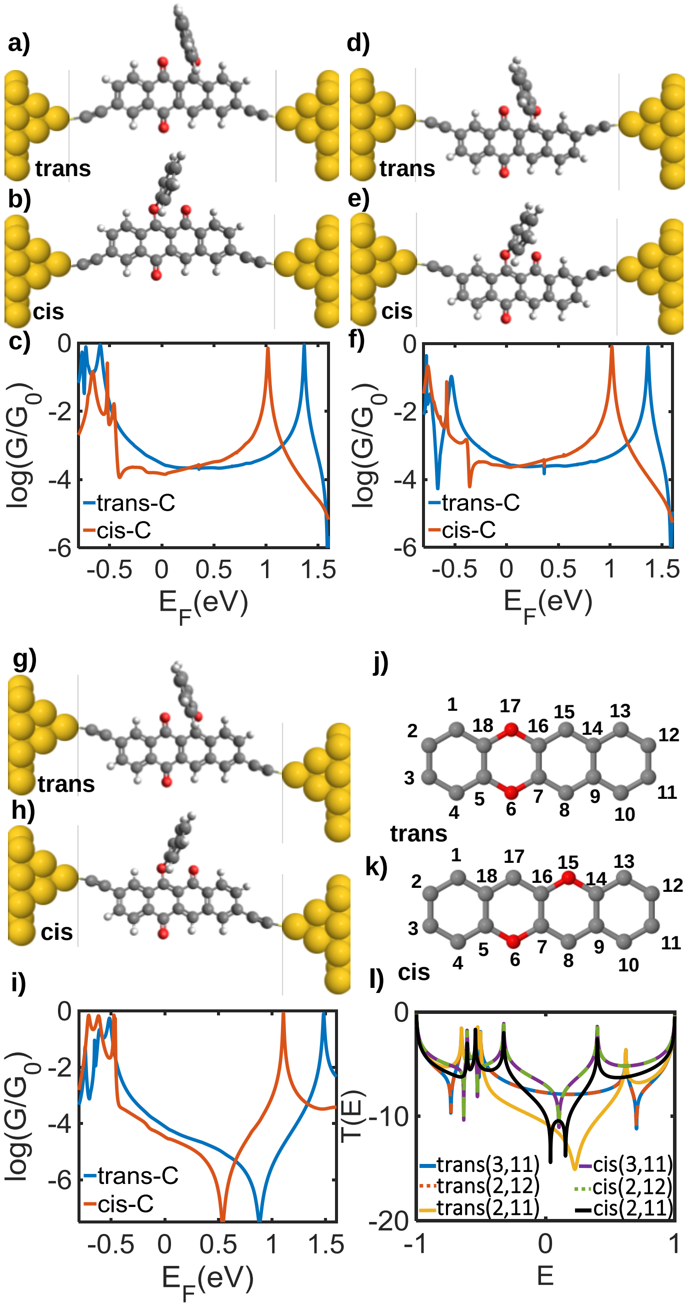

Figure S7. Molecular structure of the junctions formed phenoxyquinone isomers and electron transport through the junctions for different connectivities to electrodes using direct Au-C. (**a–f**) through *meta* connectivities (**g–j**) through *para* connectivity. (**j–l**) results obtained using simple tight binding model.
